# Aerobic De-Epoxydation of Trichothecene Mycotoxins by a Soil Bacterial Consortium Isolated Using In Situ Soil Enrichment

**DOI:** 10.3390/toxins8100277

**Published:** 2016-09-24

**Authors:** Wei-Jie He, Qing-Song Yuan, You-Bing Zhang, Mao-Wei Guo, An-Dong Gong, Jing-Bo Zhang, Ai-Bo Wu, Tao Huang, Bo Qu, He-Ping Li, Yu-Cai Liao

**Affiliations:** 1 Molecular Biotechnology Laboratory of Triticeae Crops, Huazhong Agricultural University, Wuhan 430070, China; heweijie@webmail.hzau.edu.cn (W.-J.H.); yqs198609031006@126.com (Q.-S.Y.); zybtmwx@163.com (Y.-B.Z.); guomaowei@126.com (M.-W.G.); gad-123.123@126.com (A.-D.G.); jingbozhang@mail.hzau.edu.cn (J.-B.Z.); hhttao@mail.hzau.edu.cn (T.H.); qubo@mail.hzau.edu.cn (B.Q.); 2 College of Life Science and Technology, Huazhong Agricultural University, Wuhan 430070, China; 3 College of Plant Science and Technology, Huazhong Agricultural University, Wuhan 430070, China; 4 Key Laboratory of Food Safety Research Institute for Nutritional Sciences, Shanghai Institutes for Biological Sciences, Chinese Academy of Sciences, Shanghai 200031, China; abwu@sibs.ac.cn; 5 National Center of Plant Gene Research (Wuhan), Huazhong Agricultural University, Wuhan 430070, China

**Keywords:** trichothecenes, deoxynivalenol, aerobic de-epoxydation, soil bacterium, 16S rDNA sequencing

## Abstract

Globally, the trichothecene mycotoxins deoxynivalenol (DON) and nivalenol (NIV) are among the most widely distributed mycotoxins that contaminate small grain cereals. In this study, a bacterial consortium, PGC-3, with de-epoxydation activity was isolated from soil by an in situ soil enrichment method. Screening of 14 soil samples that were sprayed with DON revealed that 4 samples were able to biotransform DON into de-epoxydized DON (dE-DON). Among these, the PGC-3 consortium showed the highest and most stable activity to biotransform DON into dE-DON and NIV into dE-NIV. PGC-3 exhibited de-epoxydation activity at a wide range of pH (5–10) and temperatures (20–37 °C) values under aerobic conditions. Sequential subculturing with a continued exposure to DON substantially reduced the microbial population diversity of this consortium. Analyses of the 16S rDNA sequences indicated that PGC-3 comprised 10 bacterial genera. Among these, one species, *Desulfitobacterium*, showed a steady increase in relative abundance, from 0.03% to 1.55% (a 52-fold increase), as higher concentrations of DON were used in the subculture media, from 0 to 500 μg/mL. This study establishes the foundation to further develop bioactive agents that can detoxify trichothecene mycotoxins in cereals and enables for the characterization of detoxifying genes and their regulation.

## 1. Introduction

The trichothecene mycotoxin deoxynivalenol (3α,7α,15-trihydroxy-12,13-epoxytrichothec-9-en-8-one, “DON”), also known as vomitoxin, is a secondary metabolite produced by many species of the genus *Fusarium* during the infection of wheat and other small grain cereals [1,2,3,4,5,6]. DON is reported to be the most frequent mycotoxin in cereal matrices worldwide [7,8,9]. DON accumulation in infected kernels renders such products unsuitable for consumption due to serious health and reproduction risks for human and farm animals [10,11,12]. The main toxic effect of DON at the cellular level in eukaryotes is associated with its ability to bind to the 60S ribosomal subunits, leading to the interference with protein translation. Ingestion of DON-contaminated feed can elicit anorexia, vomiting, low feed conversion rates, growth retardation, and immunosuppression in animals [13,14,15]. Furthermore, DON has been suggested to act as a virulence factor in infection process of *Fusarium* species [16,17,18]. Therefore, effective methods to control DON contamination are urgently needed.

Detoxification of DON by selected microorganisms represents a promising biocontrol alternative to physical and chemical methods of managing DON contamination [19,20,21]. The 12,13-epoxide group is known to be an essential part of the toxicological potency of trichothecene mycotoxins including DON and nivalenol (NIV) [21,22,23]. It has been demonstrated that the de-epoxy metabolites of DON (de-epoxy DON, dE-DON) and NIV (de-epoxy NIV, dE-NIV), which can be generated via the enzymatic reduction of the 12,13-epoxy-group to a diene, are 54-fold and 55-fold less toxic, respectively, than their parent molecules DON and NIV [22]. DON has similar cytotoxicity with NIV [22]. Many reports have been published about DON de-epoxydation by mixed bacterial cultures of ruminal and intestinal origin [24,25,26,27,28,29]. The first pure culture that was reported to de-epoxydize DON under anaerobic conditions is *Eubacterium* sp. BBSH 797, which was isolated from bovine rumen fluid [30]. Yu et al. [29] successfully isolated bacterial strains capable of transforming DON to dE-DON belonging to species of *Clostridiales*, *Anaerofilum*, *Collinsella*, and *Bacillus* from chicken digesta, which were able to modify DON into dE-DON under the condition of anaerobic incubation medium amended with 10% chicken cecal digesta extract at 37 °C. However, the de-epoxydation activities toward trichothecenes described above only occur under strict anaerobic conditions, limiting their empirical use [21]. Considering the fact that cereal grains are cultivated and stored under aerobic conditions, aerobic bacteria capable of detoxifying DON seem to hold more promises for practical applications [21,31]. To date, only one report has demonstrated DON de-epoxydation using a bacterial culture isolated under aerobic conditions [32]. The enriched bacterial culture was capable of aerobically de-epoxydizing DON at pH 6.0–7.5 and under a wide range of temperatures (12–40 °C). The partial 16S rDNA sequence analysis of the bacterial consortium revealed the presence of at least six bacterial genera, including *Serratia*, *Clostridium*, *Citrobacter*, *Enterococcus*, *Stenotrophomonas,* and *Streptomyces*. 

In this work, we describe PGC-3, a bacterial consortium with strong de-epoxydation activity on trichothecene mycotoxins under aerobic conditions, and this consortium may have an exceptional potential for developing bioactive agents to control trichothecene mycotoxins.

## 2. Results

### 2.1. Isolation of DON-Degrading Culture from Soil

**Figure 1:**
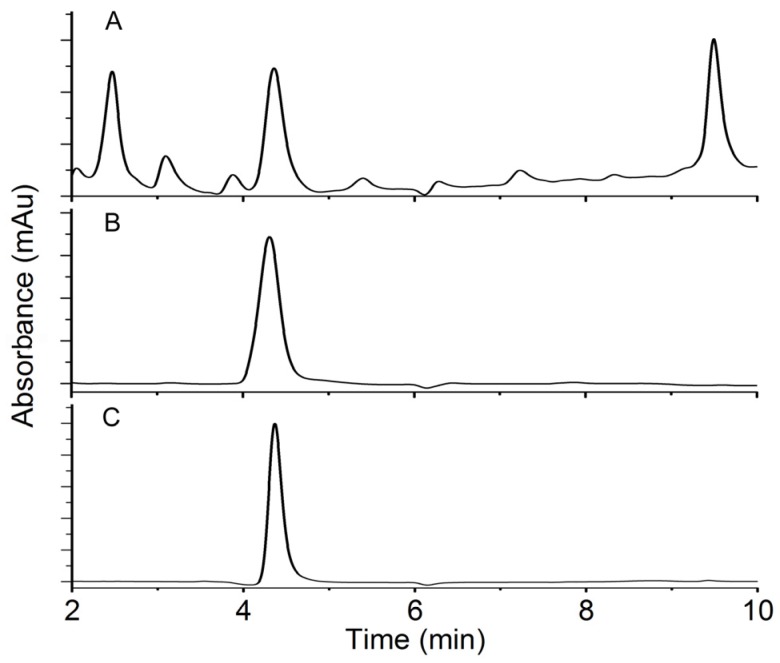
HPLC profiles of deoxynivalenol (DON) extracted from *F. graminearum* before (**A**) and after (**B**) purification, and DON purchased from Sigma-Aldrich (**C**).

DON- and water-treated (control) soil samples were screened for microorganisms capable of degrading DON. DON used for the treatments and subsequent experiments were purified from *F. gramnearum* strain 52701 and had a high purity comparable to the commercial DON from a company (Figure 1). Four of 14 samples that were screened displayed DON-degradation activity. These samples were then cultured for two cycles in MSB media containing DON. DON and its metabolites from the final subculturing of each cycle were extracted and evaluated by HPLC. The results showed that one bacterial subculture had high activity that could stably degrade DON; this culture was designated as PGC-3. No microorganisms that can degrade DON were retrieved from control samples. The PGC-3 culture was subsequently subjected to analysis in greater details. 

**Figure 2:**
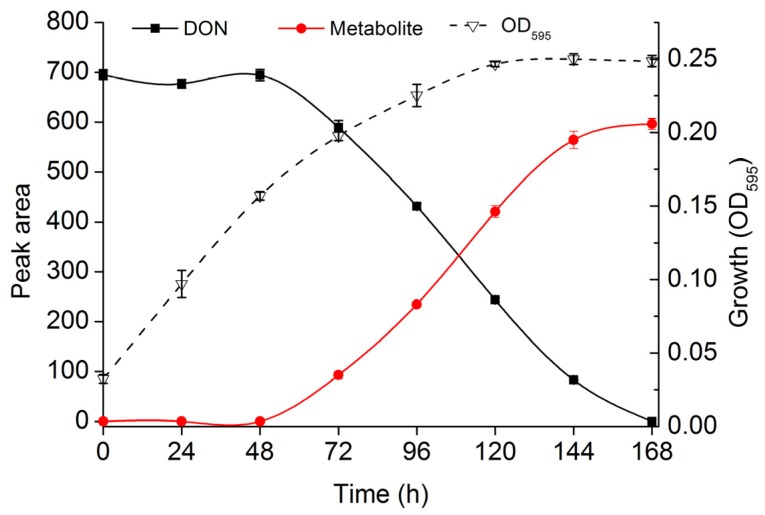
DON degradation by PGC-3 bacterial culture. DON depletion results and the new metabolite accumulation patterns were obtained in mineral salts+Bacto Peptone medium containing PGC-3 supplemented with 100 μg/mL DON over a 0–168 h time frame, in which peak areas for DON and metabolite were measured by HPLC and the bacterial growth was measured at OD_595_ at the indicated time points. The presented values are the means of three biological replicates while error bars represent standard deviations.

To further confirm this DON-degradation activity, PGC-3 was cultured in MSB medium supplemented with DON for a period of 168 h; the amount of DON and of its metabolites was measured every 24 h by HPLC. As shown in Figure 2, PGC-3 started to degrade DON after 72 h of incubation, and completely cleared DON from the medium after 168 h. Interestingly, a new DON metabolite was first detected at 72 h of incubation and the amount of this compound steadily increased thereafter, reaching a peak at the 168 h time point; bacterial growth showed a similar pattern, reaching a peak at 120 h and retaining constant levels at succedent time points (Figure 2). These results demonstrated the possibility of utilizing an in situ soil enrichment method to isolate microorganisms capable of biologically transforming DON into new metabolites. More importantly, and given our experimental conditions, these results showed that the isolated bioactive microbial culture, PGC-3, can biotransform DON under aerobic conditions.

### 2.2. Analysis of the New DON-Derived Bacterial Metabolite

**Figure 3:**
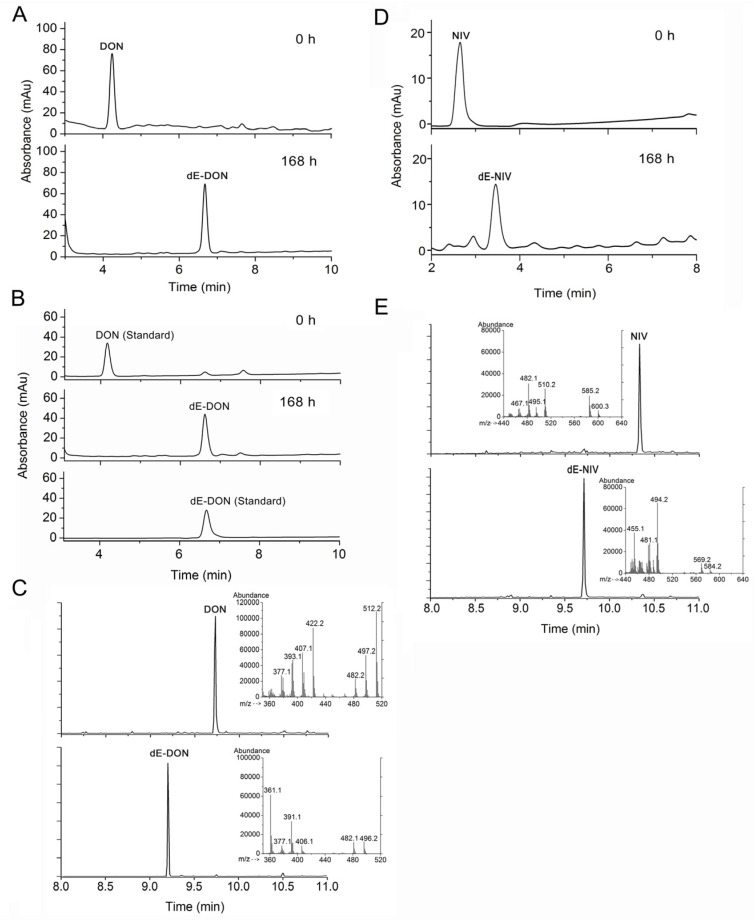
Analysis of DON, nivalenol (NIV), and their metabolites. (**A**) HPLC profiles of DON and a DON metabolite in MSB medium containing PGC-3 culture supplemented with DON purified in this study (100 μg/mL) at 0 h (upper panel) and 168 h (lower panel) of incubation; (**B**) HPLC profiles of commercial (standard) DON (upper panel) from Sigma-Aldrich at 0 h and its DON metabolite at 168 h (middle panel) of incubation in MSB medium containing PGC-3 culture, and commercial dE-DON (lower panel) from Sigma-Aldrich; (**C**) GC/MS chromatographic analysis of DON and DON metabolites. Total ion chromatograms and mass spectra of DON (upper panel) and a DON metabolite (lower panel) are shown. Detailed mass spectra of the two compounds are illustrated as small charts within the upper and lower panels; (**D**) HPLC profiles of NIV and a NIV metabolite in MSB medium supplemented with NIV and PGC-3 at 0 h (upper panel) and 168 h (lower panel) of incubation; (**E**) GC/MS chromatographic analyses of NIV and a NIV metabolite. Total ion chromatograms and mass spectra of NIV (upper panel) and a NIV metabolite (lower panel) are shown. Detailed mass spectra of the two compounds are illustrated as small charts within the upper and lower panels.

To identify the newly formed DON metabolite derived from DON during the PGC-3 culture transformation, HPLC was used to comparatively analyze extracts from the PGC-3 that were cultured in MSB containing 100 μg/mL DON for 0 h and 168 h, respectively. The obtained HPLC profiles revealed clear and different retention times of DON purified from *F. graminearum* (4.3 min) and the newly formed metabolite (6.7 min) (Figure 3A); a similar HPLC profiling pattern was seen with a commercial DON from a company and its metabolite in the same PGC-3 culture (Figure 3B). Further GC/MS profiling of derivatized DON and its metabolite(s) confirmed the difference in retention times between DON (9.732 min) and its metabolite (9.204 min). Furthermore, there was also an obvious difference in the molecular mass of these two molecules: the derivatized metabolite had a 496.2 molecular mass whereas derivatized DON was 512.2 (Figure 3C). This molecular mass difference is in accordance with atomic mass of oxygen, strongly suggesting that the DON-derived metabolite had lost one single oxygen atom. The new metabolite had the same profiling patterns in both HPLC and GC/MS analyses as dE-DON obtained from Sigma (data not shown). Taken together, these results indicate that PGC-3 was able to de-epoxydize DON into dE-DON. In parallel, nivalenol (NIV, 100 μg/mL) was spiked into PGC-3 culture and extracts after 0 h and 168 h of incubation were analyzed also by HPLC (Figure 3D) and GC/MS (Figure 3E). The results showed that PGC-3 could also de-epoxydize NIV into de-epoxy NIV (dE-NIV). Thus, PGC-3 seems to be able to de-epoxydize both of these two trichothecene mycotoxins and target the epoxy ring.

### 2.3. Effects of Temperature and pH on De-Epoxydation Activity

**Figure 4:**
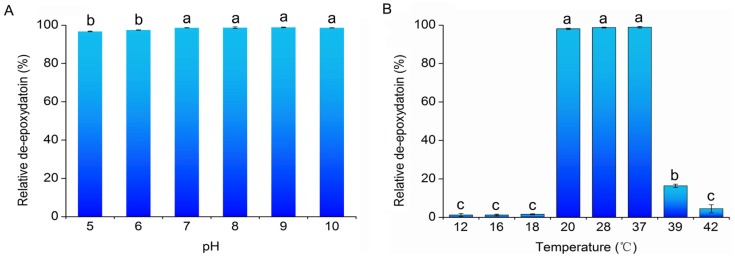
The effect of culture conditions on DON biotransformation to de-epoxy DON by PGC-3. (**A**) Effect of pH; experiments were performed in MSB at 37 °C. (**B**) Effect of temperature; experiments were performed in MSB at pH 7; microbial cultures were inoculated with DON at 100 μg/mL. The de-epoxydation activity was determined after 168 h of incubation. The values presented here reflect the means of three biological replicates. The error bars represent the standard deviations. Different characters indicate significantly difference (*p* < 0.01).

To explore the effect of temperature and pH on the de-epoxydation activity of PGC-3, cultures of PGC3 were exposed to a pH (5–10) and temperature (12–42 °C) range. DON de-epoxydation was determined by way of HPLC to determine their relative DON de-epoxydation activities. As shown in Figure 4A, PGC-3 had a constant de-epoxydation activity under all pH conditions (pH 5–10). High, consistent de-epoxydation activity was observed at temperatures ranging from 20 to 37 °C (Figure 4B). At temperatures lower than 18 °C or higher than 42 °C, PGC-3 exhibited a very low activity. These results indicate that PGC-3 retains its de-epoxydation activity under a wide range of pH and temperature conditions.

### 2.4. Bacterial Culture Complexity of PGC-3 after Subculturing under Different DON Concentrations

To enlarge the proportion of bacteria involved in DON biotransformation and to examine the bacterial population dynamics after such enrichment, PGC-3 was subcultured for 1, 7, 12, or 17 cycles under DON concentrations of 50, 50, 100, and 500 μg/mL, respectively, in four different culture cycles. The diversity and dynamic of the bacterial communities within the different cycles were analyzed by PCR–DGGE. 

**Figure 5:**
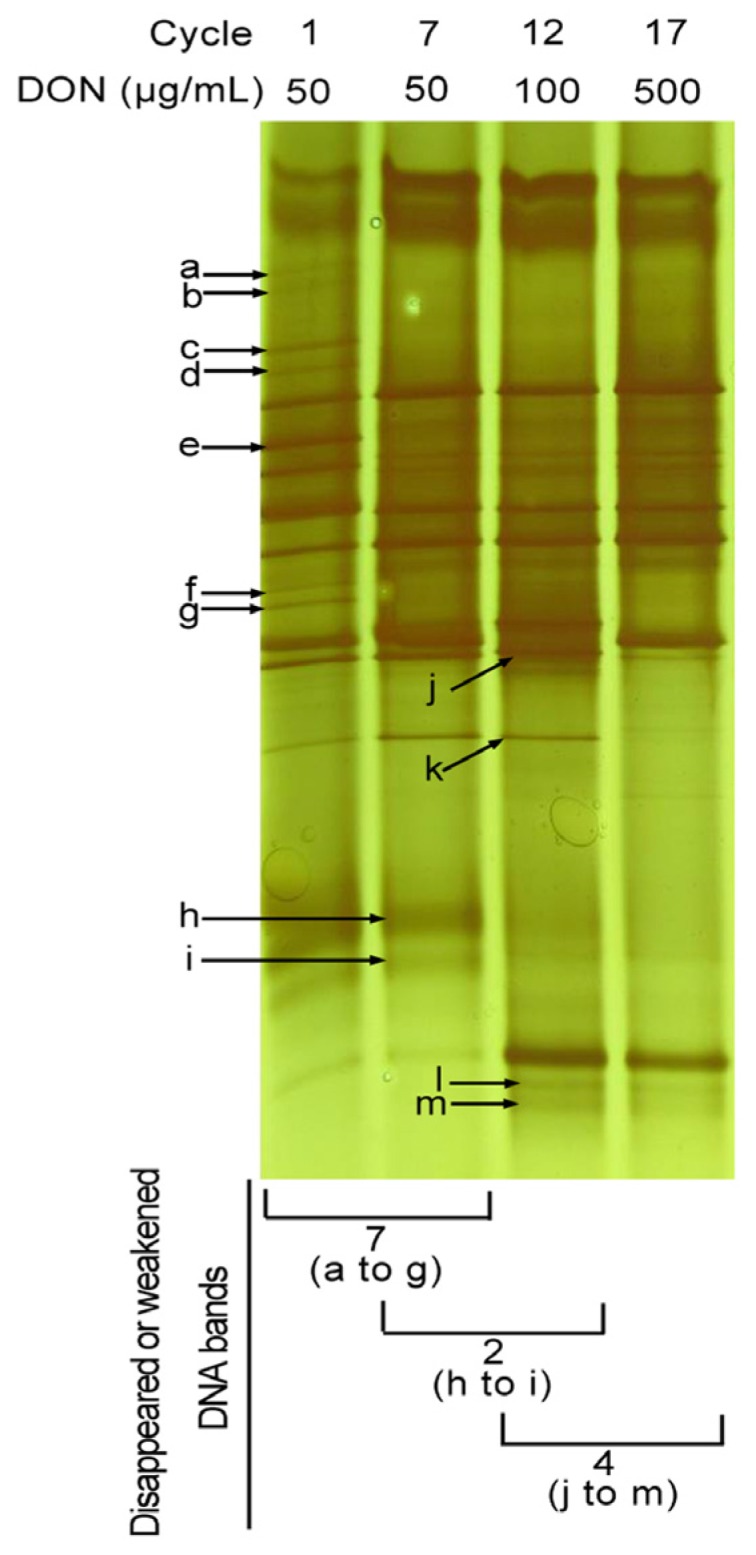
PCR-DGGE profiles of bacterial cultures after sequential subculturing under different DON concentrations. Passage/cycle numbers and DON concentrations in the utilized media are indicated above each panel. Numbers and positions of absent or weakened DNA bands in correspondence to previous enrichment step are indicated below each panel. Alphabetical letters with arrows indicate the positions of DNA bands that disappeared or (were attenuated) by the next enrichment/subculturing step.

As shown in Figure 5, the presence and absence of specific DNA bands in the DGGE gels indicated that substantial shifts in the microbial population dynamics occurred during the differential subculturing regimes. The bacterial culture tested after only one cycle (Figure 5, Lane 1) had seven more DNA bands in comparison to the one that was enriched for seven cycles (Figure 5, Lane 2). After culturing for 12 cycles (Figure 5, Lane 3), two further DNA bands disappeared (or attenuated) in comparison to bands present in the seven cycles enrichment. The bacterial culture tested after 17 cycles had the lowest number of bands, with four bands fewer than that observed at cycle 12 (Figure 5, Lane 4). These results clearly indicate that subculturing substantially reduced the population diversity of PGC-3.

### 2.5. Bacterial Culture Compositions and the Dynamics of Present Genera

**Figure 6:**
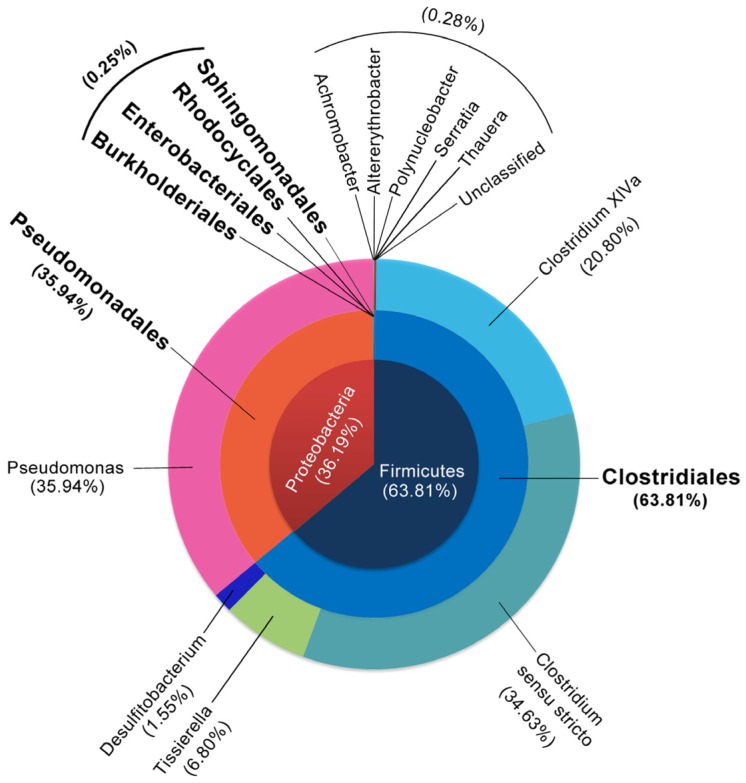
Bacterial population diversity and phylogenetic analysis. Different colors were used to reflect the bacterial distributions at the phylum (inner circle), order (middle circle), or genus levels (outer circle) within PGC-3 culture, based on operational taxonomic units (OTUs). Phylum names are given in the most inner circle. All orders and genera are labeled. Order names are given in bold while numbers in parentheses represent the percentage of the bacteria from the studied PGC-3 culture.

To investigate the population structure of PGC-3 after sequential subculturing steps, 16S rDNA sequences of the PGC3 subculture (17 cycles) on MSB medium (pH 10 and 500 μg/mL DON) were used. As shown in Figure 6, the distribution of the sequenced DNA fragments could be phylogenetically classified by phylum, order, and genus. At the phylum level, all operational taxonomic units (OTUs) belonged to two phyla, *Firmicutes* (63.81%) and *Proteobacteria* (36.19%). At the order level, a total of six orders were identified; among them, *Clostridiales* belonging to the *Firmicutes* phylum which accounted for 63.81% of PGC-3 culture, and the remaining five orders belonged to the *Proteobacteria* phylum, including *Pseudomonadales*, *Enterobacteriales*, *Burkholderiales*, *Rhodocyclales*, and *Sphingomonadales*. The *Pseudomonadales* accounted for 35.94% of the PGC-3 while the other four orders only accounted for 0.25%. At the genus level, 99.7% of the sequences were classified into 10 genera; the remaining 0.3% remained unclassified. *Pseudomonas* was the most abundant genus within the culture; accounting for 35.94%; *Clostridium* sensu strict and *Clostridium* XlVa ranked as the second (34.63%) and third (20.80%) dominant groups. The other seven genera (*Tissierella*, *Desulfitobacterium*, *Serratia*, *Achromobacter*, *Altererythrobacter*, *Polynucleobacter,* and *Thauera*) all together with the unclassified taxa, accounted for only 8.63%.

### 2.6. The Variation in Bacterial Abundance in Response to Increased DON Selective Pressure

**Figure 7:**
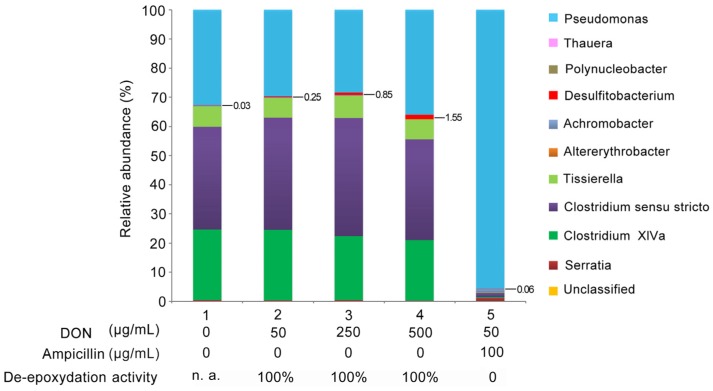
The relative abundance of bacterial genera in response to DON and ampicillin selective pressure. The bacterial relative abundance is presented in terms of percentages of the total bacterial populations per sample. The concentrations of DON and ampicillin are illustrated below each column. n.a. not applicable.

To enrich further for bacteria actively engaged in DON detoxification, PGC-3 derived from the same 16 cycles subculture was grown in MSB supplemented with different concentrations of DON. Each culture was followed up for the de-epoxydation activity individually, and the three replicates that were used for DON degradation assays were mixed together and then used for metagenomics analyses [33]. Bacterial population dynamics were analyzed by sequencing 16S rDNA to determine the relative abundance of bacterial species. One genus, *Desulfitobacterium*, displayed a clear increase in relative abundance, from 0.03% to 1.55% (52-fold increase), as the concentrations of DON were increasing from 0 to 500 μg/mL within the culture medium (Figure 7). Other bacterial genera remained constant under different DON concentrations. The bacteria from the culture containing ampicillin did not show any appreciable de-epoxydation activity at all. Within the ampicillin-supplemented culture, *Desulfitobacterium* accounted only for 0.06%, whereas the culture without any ampicillin yet containing the same concentration of DON (50 μg/mL) and showing a clear de-epoxydation activity, *Desulfitobacterium* accounted for 0.25% (a 4.2-fold difference). These results indicated that the enrichment scheme through increasing DON concentrations in subsequent culturing steps was effective and kept the selective pressure on the active bacterial species in PGC-3 culture capable of de-epoxydating DON. Furthermore, the results indicate that such species were sensitive to ampicillin presence in the culture media leading to a diminished activity. Collectively, the above results suggested that *Desulfitobacterium* was possibly a major contributor to the observed DON de-epoxydation activity.

## 3. Discussion

In this report, the in situ soil enrichment approach was highly successful for the isolation of PGC-3 culture involved in trichothecenes inactivation. Trichothecene mycotoxins such as DON and NIV are chemically very stable, yet they do not appear to accumulate in agricultural soils. This suggests that such mycotoxins are somehow biodegraded by soil organisms [21,34]. Various attempts have been made to isolate aerobic microorganisms that possess DON-degradation activities from field soil but most of such efforts were unsuccessful [21]. In this study, an in situ soil enrichment strategy was used utilizing field soils sampled from the region in China known for the occurrence of frequent *Fusarium* head blight (FHB) epidemics. DON was applied to these soil samples 12 times in a one-year span. The obtained results suggest that DON sprayed and applied as a selective pressure to these soils may indeed enrich for soil microorganisms that can catabolize DON (e.g., PGC-3). 

The obtained PGC-3 bacterial consortium has a very different population structure than the ones previously reported and was able to effectively biotransform DON into dE-DON under aerobic conditions [32]. Our results are in agreement with similar approaches that were conducted earlier using agricultural soils collected from southern Ontario, Canada [32] but with completely different culture dynamics. Ten genera of bacteria were detected thriving in PGC-3 (Figure 6). None of these genera were observed in the Canadian samples [32]. Furthermore, the Canadian study directly used 150 soil samples without enrichment to screen for DON biotransformation activities resulting in 1 soil culture that showed >99% de-epoxydation of DON [32]. In our approach, an enrichment scheme was incorporated first. The differences observed in bacterial population structures between these two DON-degrading cultures may reflect different soil ecosystems, soil compositions, and environmental conditions varying between southern Ontario (Canada) and Wuhan region (China). It is also plausible to assume that the in situ soil enrichment method that was particularly used in this study have influenced the bacterial population dynamics to a favorable outcome. 

PGC-3 exhibited consistent de-epoxydation activity at a wide range of pH (5–10) and temperature (20–37 °C) values (Figure 4), whereas the Canadian bacterial culture showed the de-epoxydation activity at a wide range of temperatures (12–40 °C) but within a relatively narrow pH range (6.0–7.5), with the most efficient DON biotransformation activities observed at pH 7.0 [32]. In addition, PGC-3 almost did not grow at 18 °C or lower temperatures and thus very low de-epoxydation activity was detected under such conditions (Figure 4B). These results further indicated that the two bacterial populations are different in their composition and dynamics and suggested that the active bacterial species that contribute to the DON de-epoxydation within the PGC-3 culture is different from the reported species observed within the Canadian culture. 

**Figure 8:**
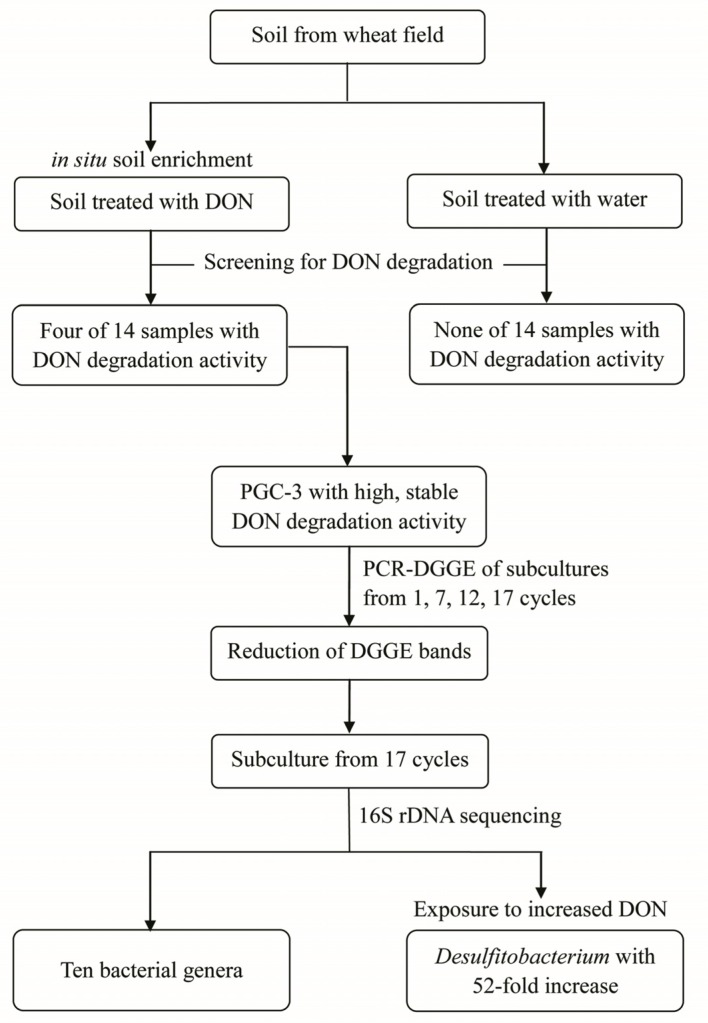
DON-degradation bacteria enrichment scheme.

The reported enrichment scheme (Figure 8) via sequential subculturing under increased DON concentrations substantially increased the relative abundance of one genus of bacteria, *Desulfitobacterium*. Ultimately, the relative abundance of *Desulfitobacterium* increased by up to 52-fold in the presence of 500 μg/mL DON in comparisons to cultures lacking DON (Figure 7). This further suggest that increasing the selective pressure associated with DON application (the frequency of application and concentration) during in situ soil enrichment schemes may further promote the natural selection of bacterial species with specific desirable phenotypes (DON de-epoxydation in this study). 

*Desulfitobacterium* spp. is a very versatile microorganism that was first isolated from bioniches contaminated with halogenated organic compounds such as soil, wastewater sludges, and freshwater sediments [35,36,37]. Most of the reported *Desulfitobacterium* strains can inactivate halogenated organic compounds; while some shows syntrophic life style coexisting with other bacterial species, which enables *Desulfitobacterium* to acquire electrons through interspecies hydrogen transfer [38]. While earlier reports classified *Desulfitobacterium* spp. as strictly anaerobic bacteria [38], the aerobic conditions applied throughout the screening and subculturing process of PGC-3, suggested that this species might be rather a facultative anaerobe. Yu et al. [29] reported that some of the anaerobic bacteria that de-epoxify DON under anaerobic conditions may be facultative aerobes, and their 16S rDNA sequences shared 99% similarity to the 16S rDNA sequence of aerobic *Bacillus arbutinivorans*. Obviously, the PGC-3 consortium that still comprised 10 bacterial genera will need to be further studied for characterization of bacteria contributing to de-epoxydation activity.

The mechanism of the destruction of the epoxide group of trichothecenes by bacteria remains an open question. Various commercial epoxide hydrolases or organisms with known epoxide hydrolase activity did not show any de-epoxydation of DON [39], and trichothecenes were observed to be resistant to epoxide hydrolase from rat liver [40]; these results suggest that enzymatic hydrolysis is unlikely the mode of de-epoxydation. Thus, it is speculated that enzymatically reductive de-epoxydation of trichothecenes may likely be the action mode used by bacteria [21,41] that may use the energy derived from medium (such as peptone) for the proposed reductive reaction. Further investigation of the PGC-3 through metatranscriptomics and metaproteomics may provide insight into the mechanism underlying the de-epoxydation of trichothecenes. 

*Clostridium sensus* *trictu* and *Tissierella* that are considered as obligated anaerobic bacteria were present in PGC-3 consortium (Figure 6 and Figure 7). Previously, Islam et al. also identified obligate/facultative anaerobic bacteria such as *Clostridium*, *Citrobacter*, *Serratia,* and *Enterococcus* under aerobic conditions [32]. It is likely that some species from these bacterial genera may have evolved facultative features. Further screening and investigation will provide more information on the molecular and phylogenetic bases of these bacteria. 

It should be noted that some species from *Clostridium* genus, such as *Clostridium difficile,* are considered to be one of the significant causes of health care-associated infections [42], whereas other members from this genus may help in the treatment of diseases such as cancer, as those *Clostridium* can selectively target cancer cells. Thus, *Clostridium* could be used to deliver therapeutic proteins to tumors [43]. A similar phenomenon was reported for *Pseudomonas* genus; *P. aeruginosa* is an opportunistic human pathogen [44], and *P. syringae* is a plant pathogen [45]. On the other hand, some members of *Pseudomonas* genus are able to metabolize chemical pollutants in the environment. For example, *P. alcaligenes* can degrade polycyclic aromatic hydrocarbons [46]. 

Analyses of cytotoxicity and transcriptomics of human intestinal epithelial cells treated with DON and dE-DON revealed that dE-DON did not change the oxygen consumption or impair the barrier function and were thus not cytotoxic; there were no differentially expressed genes between control and dE-DON-treated cells, whereas 323 genes were differentially expressed between DON-treated and control cells [23]. As for the cell toxicity of dE-NIV, a 5-bromo-20-deoxyuridine incorporation assay assessing DNA synthesis showed that dE-NIV was 55 times less toxic than NIV [22]. These molecular and cellular studies verify the previous findings that de-epoxydation of trichothecenes is a significant biological detoxification reaction based on pathophysiologic and toxicologic studies [14,25,47,48,49]. 

In DON detoxification applications, anaerobic bacteria have a limited range for practical applications which mainly confined to the digestive systems of farm animals. Aerobic bacteria would much more favored for the decontamination of trichothecene mycotoxins in cereal grains, as such bacteria can grow in agricultural fields and storage silos under less restrictive aerobic conditions [31]. This study demonstrates the existence and the potential for an aerobic de-epoxydation of DON and NIV by a bacterial consortium obtained from natural soil habitant by utilizing efficient and novel enrichment scheme. The obtained bacterial consortium has the capacity to serve as a base to further develop practical DON detoxifying agents for food/feed applications or utilized as a source to fish for enzymatic solutions to address DON bio-toxicity.

## 4. Materials and Methods 

### 4.1. Trichothecenes and Culture Media

DON from *F. graminearum* 52701 [10] and NIV from *F. graminearum* 54606 [50] was prepared as described earlier by Clifford et al. [51]. Briefly, BOND ELUT SPE columns (Varian, Palo Alto, CA, USA) and semi-preparative HPLC (Agilent Technologies, Santa Clara, CA, USA) were used for purification of DON. Standard samples of DON and de-epoxy DON (dE-DON) were purchased from Sigma-Aldrich (St. Louis, MO, USA). MSB (mineral salts with Bacto Peptone) medium [32], supplemented with DON, was used to screen for microbial cultures for DON-biotransformation capabilities.

### 4.2. Soil Samples

Soil samples were randomly collected from wheat fields at Huazhong Agricultural University in Wuhan, China. To enrich for microorganisms with DON-biotransformation activity, the 14 soil samples were mixed with wheat spikelets infected by *F. graminearum*, divided into pots (10 cm inner diameter and an 8 cm height), and placed in a plant growth chamber for 12 months. Aliquots (10 mL) of DON solution (20 μg/mL in water) were sprayed onto the mixtures (in the pots) once every month. In a parallel control, the same 14 soil samples were sprayed with water under the same conditions. The samples (soils treated with DON and water for 12 times over a one-year period) were later used to isolate microorganisms with DON-biotransformation capabilities.

### 4.3. Screening of Soil Samples

Soil samples (0.5 g) treated with DON or water in growth chambers were suspended in 2 mL of MSB medium supplemented with 50 μg/mL of DON. In total, 28 samples were screened: there were 14 samples each from DON-treated or water-treated soils. The suspended soil samples were incubated at 28 °C for seven days and shaking at 220 rpm. DON in the culture media was extracted, and DON concentrations were monitored by HPLC as described below. Culture samples that showed decreased concentrations of DON were selected for further analysis. Aliquots (100 μL) of the culture samples were transferred to 1 mL of fresh MSB medium containing DON (50 μg/mL) and cultured for additional seven days under the same conditions; DON concentrations from the culture samples were further measured by HPLC. This procedure was repeated twice to confirm the observed DON degradation activity.

### 4.4. Extraction and Analysis of Trichothecenes and Their Metabolites

The extraction of trichothecene mycotoxins in addition to the final products of biotransformation in the above bacterial cultures was carried out as described previously [52]. An aliquot (100 μL) of a fresh culture was mixed with 300 μL of ethyl acetate. After vigorous shaking for 10 min, the resultant organic solvent layer was collected. This procedure was repeated three times, and the collected organic solvent layer was dried by evaporation. The residue was resuspended in 100 μL of methanol. The prepared extracts were analyzed as described previously [27], with some modifications. A 10 μL aliquot was injected into an HPLC system consisting of a 1260 infinity quaternary pump, a 1260 Infinity UV detector, an Agilent Zorbax Eclipse XDB-C18 column (4.6 × 150 mm, 5 μm); Agilent ChemStation software (B.04.03) was used to implement the method and analyze the results (Agilent Technologies, Santa Clara, CA, USA). The column temperature was kept to 30 °C. The elution flow rate was 1 mL/min with the following methanol–water linear gradient: ramp from 25% methanol to 75% over 15 min, ramp to 80% methanol over 5 min, hold for 3 min, then decrease back to 25% over 3 min, and hold for 3 min. Trichothecene mycotoxins and their metabolites were measured at the 218 nm wavelength. All biotransformation assays were performed in triplicate.

Alternatively, the purified trichothecene mycotoxins and their metabolites were analyzed by gas chromatography/mass spectrometry (GC/MS) using an Agilent 7890A gas chromatograph (Agilent Technologies, Santa Clara, CA, USA) equipped with an Agilent 5975C MSD and a 30 m capillary column of DB-5MS (J & W Scientific, Santa Clara, CA, USA, 0.25 mm ID, 0.25 μm film thickness) as previously described [53], with the following modifications: the GC conditions were as follows: carrier gas helium, flow rate 1 mL/min, the column was initially held at 80 °C for 1 min and then increased to 280 °C at 25 °C/min and held at this temperature for 6 min. The MS conditions were as follows: electron ionization (EI) mode, full scan, ionization energy 70 eV, mass range *m*/*z* 100–600 for DON and its metabolites, and *m*/*z* range from 100 to 700 for NIV and its metabolites.

### 4.5. Temperature and pH Conditions Used for DON De-Epoxydation

DON de-epoxydation ability of the enriched microbial culture was investigated under different temperatures and pH conditions. Enriched microbial culture (100 μL) aliquots were added to 1 mL of various media. The effect of temperature was studied by incubating the culture in MSB medium (pH 7) at 12, 20, 28, 37, and 42 °C. To determine the effects of pH, the pH of the MSB medium was adjusted to 5.0, 6.0, 7.0, 8.0, 9.0, and 10.0. Media with different pH values were incubated at 37 °C. The cultures were supplemented with 100 μg/mL DON before incubations. Triplicates were used for each temperature and pH condition. DON de-epoxydation activity was calculated with this formula: Reduction in DON concentration (%) = (Concentration of DON original − Concentration of DON residual)/Concentration of DON original × 100.

### 4.6. Genomic DNA Extraction

Total genomic DNA of each sample was extracted using an AxyPrepTM Bacterial Genomic DNA Miniprep Kit (Axygen Biosciences, Union, CA, USA) according to the manufacturer’s instructions. Extracted genomic DNA was stored at −70 °C until use.

### 4.7. PCR-DGGE Analysis

Bacterial 16S rDNA sequences were amplified using the following primers: EUB968-GC- for (5′-**CGCCCGGGGCGCGCCCCGGGCGGGGCGGGGCAGGGG**AACGCGAAGAACCTTAC-3′; the GC clamp is in bold) and EUB-L1401-rev (5′-CGGTGTGTACAAGACCC-3′) [54]. Each PCR reaction mix contained 1× PCR buffer, 0.2 mM dNTP mix, 1 μM of each primer, 2.5 U Taq DNA polymerase (TransGen Biotech, Beijing, China), and 100 ng of template DNA. PCR amplifications were carried out in a final volume of 50 μL. The thermocylcing conditions were as follows: initial denaturation for 5 min at 94 °C; 35 cycles of 30 s at 94 °C, 30 s at 60 °C, and 1 min at 72 °C; and a final extension for 10 min at 72 °C. The DGGE analysis of PCR amplicons was performed using Bio-Rad DCode Universal Mutation Detection System (Bio-Rad, Hercules, CA, USA) as described previously [29]. The amplicons were separated in 8% polyacrylamide (acrylamide/bisacrylamide 37.5:1) gels containing a 40%–60% gradient of urea and formamide. The denaturing solution used consisted of 7 M urea and 40% (*v*:*v*) deionized formamide. DNA bands were visualized by silver staining. The numbers of DNA bands, including the presence and density, were used to judge the complexity of bacterial cultures [29].

### 4.8. Analysis of Bacterial Population and Their Representing Species

A primer pair targeting the V3 and V4 regions of 16S rDNA genes was selected according to Klindworth et al. [55]. Illumina adapter overhang nucleotide sequences were added to the primers. The full length primer sequences were P1 (5′-TCGTCGGCAGCGTCAGATGTGTATAAGAGACAGCCTACGGGNGGCWGCAG-3′) and P2 (5′-GTCTCGTGGGCTCGGAGATGTGTATAAGAGACAGGACTACHVGGGTATCTAATCC-3′). Each sample was amplified under the following conditions: 95 °C for 3 min; 25 cycles of 95 °C for 30 s, 55 °C for 30 s, followed by 72 °C for 30 s, with a final extension 72 °C for 5 min. The 16S V3 and V4 amplicons were purified, and a subsequent limit-cycle amplification step was performed to add Illumina sequencing adapters and dual-index barcodes to the amplicon targets. The libraries were normalized and pooled then sequenced using paired-end method by Illumina MiSeq at the Shanghai Bio. Tech. Company (Shanghai, China). Raw fastq files were demultiplexed and quality-filtered using the FASTX Toolkit (version 0.0.13) [56]. Reads of low quality were discarded and trimmed from the dataset based on previously-described criteria [57]. Operational Taxonomic Units (OTUs) were clustered at 97% similarity, and the taxon abundance of each sample was generated via the RDP database using QIIME (Quantitative Insights Into Microbial Ecology) software (version 1.9.0, University of Colorado, Boulder, CO, USA, [58]).

### 4.9. Bacterial Sub-Culturing under Diverse Growth Conditions

PGC-3 was sequentially subcultured under four different conditions to enrich for the proportion with active bacteria (in regard to DON detoxification): (i) One cycle culture: MSB with 50 μg/mL DON, pH 10, was used for one cycle of subculturing. Each cycle lasted seven days, and 100 μL of the culture from the previous cycle was passed to 1 mL of fresh MSB medium for the next generation; (ii) Seven cycles culture: the same culture conditions detailed for (ii) was used sequentially for six cycles; (iii) 12 cycles culture: MSB with 100 μg/mL DON, pH 10, was used for sub-culturing bacteria for five sequential cycles; (iv) 17 cycles culture: MSB with 500 μg/mL DON, pH 10, was used for subculturing up to an additional five sequential cycles. The bacterial samples from the above four culture conditions were comparatively assayed by PCR-DGGE.

To enrich further for the active bacteria, the PGC-3 culture that was passed for 16 cycles as mentioned above was grown later in MSB medium, pH 10, supplemented with varying concentrations of DON (0, 50, 250, and 500 μg/mL), or with a fixed concentration of DON (50 μg/mL) plus ampicillin (100 μg/mL), for one cycle. The bacterial samples from these five cultures were comparatively analyzed for the de-epoxydation activity of DON by HPLC while the relative abundance of various bacterial species was analyzed by 16S rDNA sequencing of the entire bacterial community.

### 4.10. Statistical Analysis

All the results were analyzed using ANOVA for multiple comparisons followed by the Duncan test using SAS software v.8.1 (SAS institute, Cary, NC, USA), using significance levels of 0.01.
